# Automatic recognition of murmurs of ventricular septal defect using convolutional recurrent neural networks with temporal attentive pooling

**DOI:** 10.1038/s41598-020-77994-z

**Published:** 2020-12-11

**Authors:** Jou-Kou Wang, Yun-Fan Chang, Kun-Hsi Tsai, Wei-Chien Wang, Chang-Yen Tsai, Chui-Hsuan Cheng, Yu Tsao

**Affiliations:** 1 National Taiwan University Children’s Hospital, Taipei, Taiwan; 2 iMediPlus Inc., Hsinchu, Taiwan; 3 Research Center for Information Technology Innovation at Academia Sinica, Taipei, Taiwan

**Keywords:** Cardiovascular diseases, Cardiology, Diseases, Health care, Diagnosis, Software

## Abstract

Recognizing specific heart sound patterns is important for the diagnosis of structural heart diseases. However, the correct recognition of heart murmur depends largely on clinical experience. Accurately identifying abnormal heart sound patterns is challenging for young and inexperienced clinicians. This study is aimed at the development of a novel algorithm that can automatically recognize systolic murmurs in patients with ventricular septal defects (VSDs). Heart sounds from 51 subjects with VSDs and 25 subjects without a significant heart malformation were obtained in this study. Subsequently, the soundtracks were divided into different training and testing sets to establish the recognition system and evaluate the performance. The automatic murmur recognition system was based on a novel temporal attentive pooling-convolutional recurrent neural network (TAP-CRNN) model. On analyzing the performance using the test data that comprised 178 VSD heart sounds and 60 normal heart sounds, a sensitivity rate of 96.0% was obtained along with a specificity of 96.7%. When analyzing the heart sounds recorded in the second aortic and tricuspid areas, both the sensitivity and specificity were 100%. We demonstrated that the proposed TAP-CRNN system can accurately recognize the systolic murmurs of VSD patients, showing promising potential for the development of software for classifying the heart murmurs of several other structural heart diseases.

## Introduction

Ventricular septal defect (VSD), a type of congenital heart disease (CHD) caused by developmental defects of the interventricular septum, is the most common type of heart malformation present at birth. It occurs in approximately 2–6 of every 1000 live births and accounts for approximately 30% of all CHDs in children/adolescents^1–4^. The clinical presentation of a VSD is correlated with the size of the defect^5^. Mild VSDs are usually asymptomatic and commonly occur spontaneously within close proximity^6^. Patients with medium defects often suffer from dyspnea. Patients with severe VSDs exhibit cyanosis, dyspnea, syncope, or heart failure and require adequate surgeries unless the defects spontaneously decrease^7–9^. VSDs can also be classified according to the morphology and anatomical location of the defect. They can also be classified into four anatomical types: type I (outlet supracristal, subarterial, or infundibular), type II (perimembranous, paramembranous, or conoventricular), type III (inlet, atrioventricular canal, or atrioventricular septal defect), and type IV (muscular or trabecular)^10–12^. The perimembranous type is the most common (~ 80%), followed by the muscular (15–20%), inlet (~ 5%), and outlet (~ 5%) types.

Similar to many other heart malformations, heart murmurs can be heard in patients with VSD^13^. Patients with a VSD are known to commonly experience holosystolic murmurs, owing to the turbulence of the blood flow between the left and right ventricles^14,15^. Murmur recognition with auscultation is conventionally used for the screening and diagnosis of VSD^16^. However, the accuracy of this method largely depends on clinical experience and is a challenge for most young and inexperienced clinicians^17^. Therefore, the development of tools to automatically recognize heart-sound patterns can help physicians diagnose heart disease.

Artificial intelligence has recently been widely used in computer-aided diagnosis^18,19^. For example, many algorithms that claim to automatically recognize and classify medical images have been developed using deep learning^20–22^. Recent efforts have shown significant advances using artificial neural networks (ANNs) or deep neural networks (DNNs) to detect and classify heart sounds^23–25^. Convolutional neural networks (CNNs) have also been used to identify heart murmurs^26^. The aim of this study was to develop a novel algorithm that can automatically recognize the systolic murmurs of VSD patients using a novel temporal attentive pooling–convolutional recurrent neural network (TAP-CRNN) model^27^.

## Results

Heart sounds from 76 subjects, including 51 VSD patients and 25 patients without significant heart malformations, were included in this study. Table 1 shows the mean age, height, weight, and sex distribution of these subjects. There were no statistically significant differences between the group suffering from VSD and the normal group, with regard to these clinical variables. Regarding the types of VSDs, most patients were diagnosed with the type 2 VSD (perimembranous type) and a minor VSD. The details of the VSD types are listed in Table 2.

**Table 1:** Basic information of the subjects in this study.

Variables	VSD group (N = 51)	Normal group (N = 25)
Age (years)	22.12 ± 16.96 (min: 2; max: 65)	29.30 ± 18.67 (min: 4.3; max: 65)
**Sex**		
Male [n; (%)]	30 (58.82%)	14 (56%)
Female [n; (%)]	21 (41.18%)	11 (44%)
Height (cm)	147.23 ± 27.86	155.04 ± 23.91
Weight (kg)	46.75 ± 22.70	55.82 ± 24.63

**Table 2:** Details of the VSD types included in this study.

VSD types (N = 51)	Case number (%)
Type I: infundibular, outlet	2 (3.92%)
Type II: perimembranous	42 (82.35%)
Type III: inlet, atrioventricular	0
Type IV: muscular, trabecular	5 (9.80%)
Unknown	2 (3.92%)
Size classification*	
Small	30 (58.82%)
Medium	13 (25.49%)
Large	4 (7.84%)
Unknown	4 (7.84%)

*Small VSD: Qp/Qs < 1.5; medium VSD: 1.5≦Qp/Qs < 2; large VSD: 2≦Qp/Qs; where Qp indicates pulmonary blood flow, Qs indicates systemic blood flow.

Two repeated heart sound recordings were obtained at each of the five standard auscultation spaces. For some subjects whose recordings did not qualify, owing to the presence of noise, more than two recordings were obtained within the same auscultation space to confirm the quality of the soundtracks. A total of 776 heart soundtracks were recorded from 76 subjects, including 525 soundtracks from VSD patients and 251 soundtracks from normal subjects. The number of soundtracks in the training and test sets is shown in Table 3.

**Table 3:** Number of subjects and heart sound recordings in this study.

Variables	VSD group	Normal group
Number of subjects	51	25
Number of sound recordings	525	251

**Figure 1:**
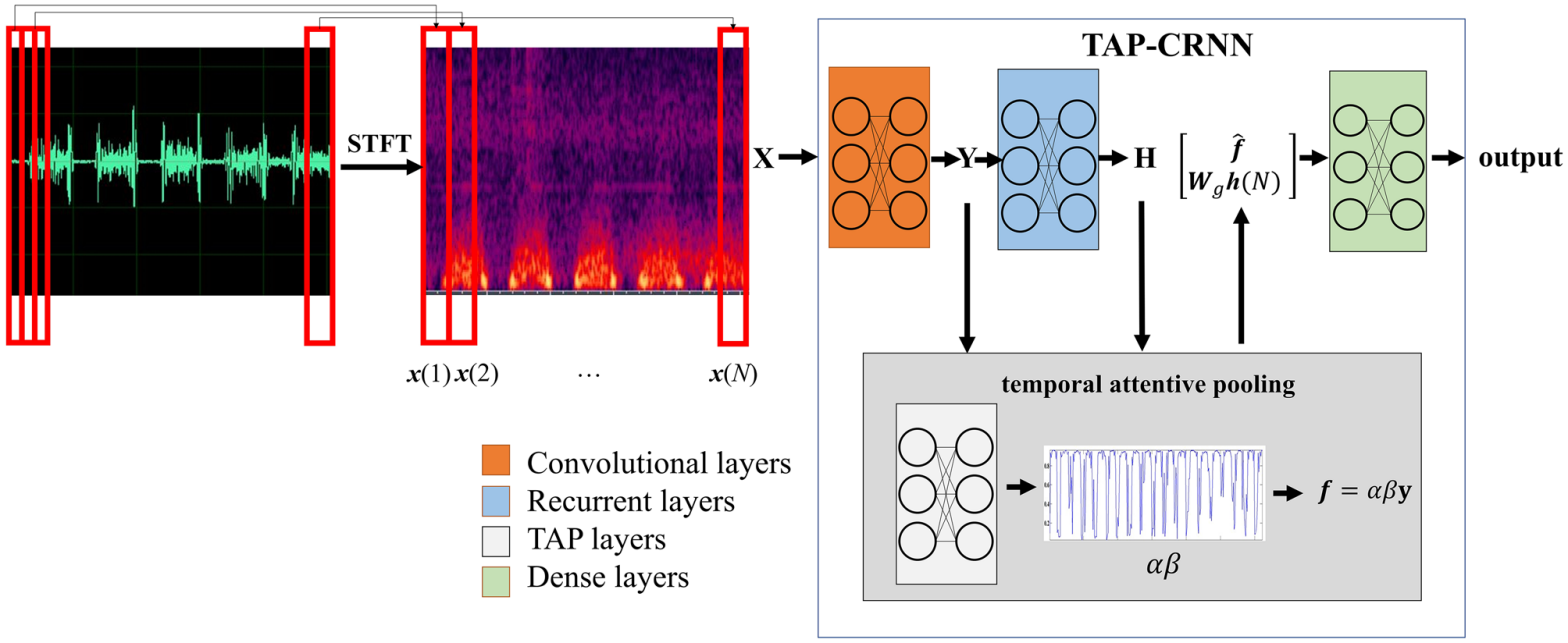
The structure of the TAP-CRNN model. STFT was used to transform the phonocardiogram (PPG) signals to spectral features at the first step. The second step used CNN to extract invariant spatial–temporal representations from the spectral features. Then RNN was used to extract long temporal-context information in the representations for classification in the following step. Finally, TAP was used to assign importance weights for each frame in the systolic regions in the fourth step. STFT: short time fast Fourier transformation; LSTM: long-short term memory; TAP: temporal attentive pooling.

The TAP-CRNN model was used to recognize systolic murmurs in the current study. The structure of TAP-CRNN is described in Fig. 1, in which the phonocardiogram (PCG) signals were first converted into the spectral domain using a short-time Fourier transform, with a frame length of 512 and a frameshift of 256. Then, each frame of PCG signals is represented by a 257-dimentional log-power spectral feature vector. An input signal was classified as a systolic murmur or a normal signal for training the TAP-CRNN model, which consists of four parts: convolutional, recurrent, temporal attentive pooling (TAP), and dense layers. The convolutional layers extracted invariant spatial–temporal representations from the spectral features. The recurrent layers were used in the following step to extract the long temporal context information from the representations. The TAP layers were then used to assign importance weights to each frame in the systolic regions. Finally, the classified results were generated by the dense layers according to the temporal attentive pooling feature outputted from the TAP layers.

**Figure 2:**
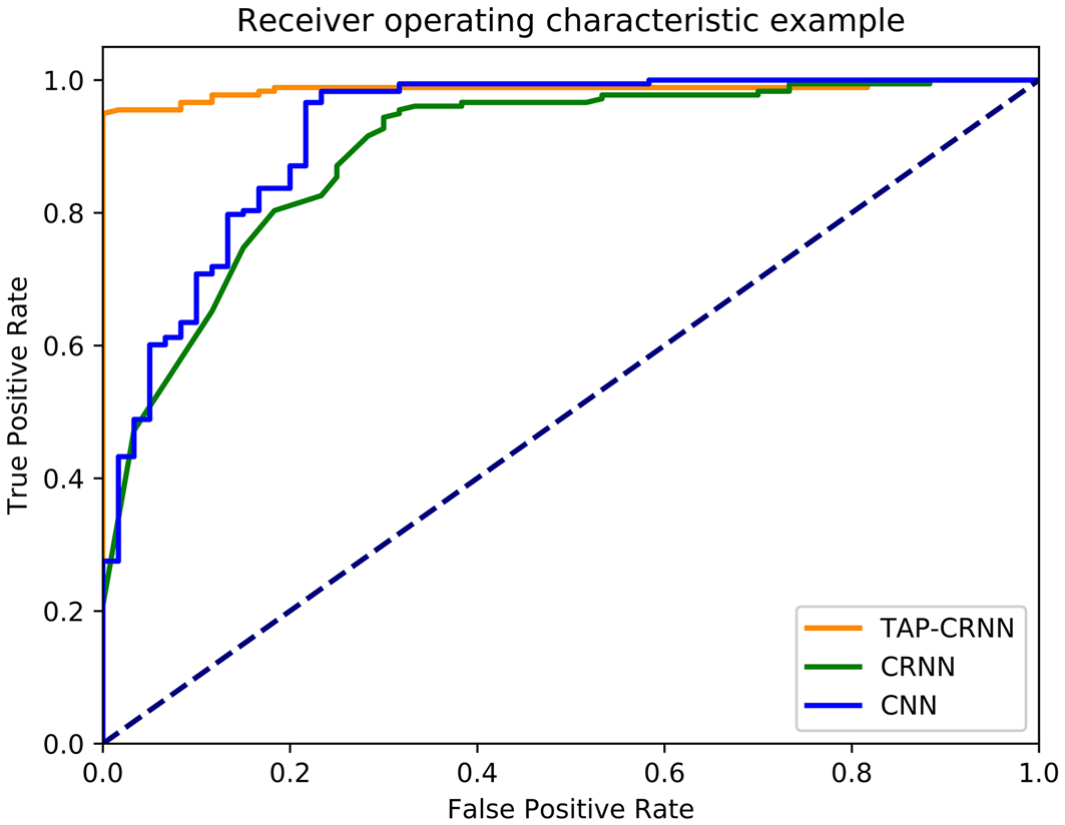
The experimental result of the ROC curves of the CNN, CRNN, and TAP-CRNN models.

The performance of the algorithm for systolic murmur recognition was analyzed for two different tasks, namely, a train–test split and K-fold cross-validation. Table 4 shows the performance of TAP-CRNN for systolic murmur recognition in the train–test split task. The CNN and CRNN models for systolic murmur recognition were also analyzed for comparison. The CNN model comprised three convolutional layers, where the first layer consisted of 32 filters with a kernel size of 1 × 4, the second layer included 32 filters with a kernel size of 1 × 4, and the third layer contained 32 filters with a kernel size of 4 × 4; the model also comprised two dense layers, with each layer composed of 512 neurons. The CRNN model comprised two convolutional layers, with each layer consisting of 16 filters with a kernel size of 1 × 4; two recurrent layers (long short-term memory unit), with each layer including 256 neurons; and two dense layers, with each layer containing 256 neurons. Compared with the CRNN architecture, a TAP-CRNN comprises an additional TAP layer. The hyperbolic tangent units were used in all the models, and the softmax unit was used in the last output layer. Adaptive moment estimation (Adam)^28^ was used as the optimizer. For the train–test split task, the entire set of data were divided into 70% (191 normal sounds, and 351 systolic murmur sounds) and 30% (60 normal sounds and 178 systolic murmur sounds) for training the murmur recognition models and testing their performances, respectively. For this task, the sensitivity and specificity scores were 88% and 85% for CNN, 92% and 93% for CRNN, and 97% and 98% for TAP-CRNN, respectively. In supplementary Tables 1–3, 2 × 2 tables of positive and negative events are shown. The receiver operating characteristic (ROC) curves of CNN, CRNN, and TAP-CRNN are shown in Fig. 2. The results show that the use of the TAP-CRNN model achieves a better accuracy for systolic murmur recognition when compared to the use of the CNN and CRNN models. The K-fold cross-validation task was used to further verify the reliability of the system performance. We conducted experiments using a fourfold (K = 4) setup. We first divided the entire set of PCG data into four groups, and roughly equal numbers of VSD patients and normal people were assigned to each group. We used data belonging to three out of these four groups for training the TAP-CRNN model, and the remaining group was used for testing. There were no overlapping subjects in the training and test sets. We carried out this procedure four times, the results of which have been listed in Table 5. From Table 5, we can see that the fourfold results are quite consistent and share the same trends as the results reported in Table 4 (the train– test split task). The average sensitivity and specificity scores over 4-folds were 97.18% and 91.98% of TAP-CRNN, confirming that the proposed TAP-CRNN can reliably produce satisfactory results for all evaluation metrics.

**Table 4:** Results of testing the algorithm’s ability to distinguish systolic murmurs from normal heart sounds.

	Accuracy	Sensitivity	Specificity	PPV	NPV
CNN	87.0%	87.6%	85.0%	94.5%	69.9%
CRNN	92.0%	91.6%	93.3%	97.6%	78.9%
TAP-CRNN	97.1%	96.6%	98.3%	99.4%	90.1%

**Table 5:** Results of fourfold cross validation of TAP-CRNN.

	Accuracy	Sensitivity	Specificity	PPV	NPV
1st fold	98.4	99.2%	96.7%	98.5%	98.3%
2nd fold	96.8	96.2%	98.3%	99.2%	92.2%
3rd fold	96.4	99.3%	90.0%	95.7%	98.2%
4th fold	90.2	94.0%	82.9%	91.2%	87.9%
Average	95.45	97.18	91.98	96.15%	94.15%

The capability of the TAP-CRNN model for recognizing the systolic murmurs at the five standard auscultation sites was also analyzed (Table 6, supplemental Tables 4–8). Both the second aortic and the tricuspid areas showed 100% sensitivity and 100% specificity. The sensitivity was decreased in the other spaces, including the aortic (95.5%), pulmonic (94.1%), and mitral (94.1%) areas.

**Table 6:** Test results of the TAP-CRNN model’s ability to distinguish systolic murmur from normal heart sounds at the 5 standard auscultation locations.

Auscultation area	Accuracy	Sensitivity	Specificity	PPV	NPV
Aortic area	94.6%	95.5%	91.7%	97.7%	84.6%
Pulmonic area	95.7%	94.1%	100%	100%	85.7%
Second aortic area/ Erb’s point	100%	100%	100%	100%	100%
Tricuspid area	100%	100%	100%	100%	100%
Mitral area/apex	95.7%	94.1%	100%	100%	85.7%

## Discussion

A murmur is a sound generated by the turbulent blood flow in the heart. Under normal conditions, the blood flow in a vascular bed is smooth and silent. However, blood flow can be turbulent and produce extra noise when the heart has a structural defect^29^. Murmurs can be classified based on their timing, duration, intensity, pitch, and shape. Specific murmur patterns may occur as a result of many types of structural heart diseases^14^. For example, holosystolic murmurs, which are characterized by uniform intensity during the systolic period, usually appear in patients with mitral regurgitation (MR), tricuspid regurgitation (TR), or VSD^30–32^. Murmurs that occur during the systolic period with a crescendo-decrescendo shape are called systolic ejection murmurs and are often heard in patients with aortic stenosis (AS), pulmonic stenosis (PS), and atrial septal defect (ASD)^30^. Experienced cardiologists may successfully distinguish these specific heart sound patterns during routine auscultation, and this capability is important in disease diagnosis. However, it is always a challenge for young and inexperienced physicians to make a correct diagnosis based on auscultation^17,33^. Therefore, the development of tools that can automatically classify specific murmur types is necessary and clinically significant^34,35^.

In recent years, CNNs have been widely used in computer-aided diagnosis^36,37^. Previous studies have used a CNN to classify pathological heart sounds^38,39^. A recurrent neural network is another model frequently used in computer-aided diagnosis^40,41^. In this study, we combined CNN and RNN models (forming a CRNN model) to recognize the systolic murmurs from VSD patients. We used a convolutional unit to extract invariant spatial–temporal representations and the recurrent unit to capture long temporal-context information for systolic murmur recognition. In addition, the TAP mechanism was also applied in the CRNN model to assign an importance weight for each frame within the murmur regions. Finally, the overall model is called TAP-CRNN. From our experimental results, the TAP-CRNN model demonstrated an accuracy of 96% for distinguishing systolic murmurs from normal heart sounds, outperforming both CNN and CRNN without TAP.

For heart sounds recorded in the tricuspid and second aortic areas (Erb’s point), both the sensitivity and specificity reached 100% when the TAP-CRNN model was used. A high accuracy in these two areas is reasonable because the murmurs caused by the blood flow between the right and left ventricles can be most clearly heard in the tricuspid area or the lower left sternal border, which overlies the defect^42^.

The intensity of the murmur is inversely proportional to the size of the VSD. The ability of the algorithm to recognize the murmurs caused by a moderate or large VSD was also tested in the current study. In the test set, 63 soundtracks from 6 patients with moderate/large VSDs were included. When using the TAP-CRNN model, the murmurs of these soundtracks from moderate/large VSDs can be accurately recognized, except for two soundtracks recorded in the mitral area. Although the results obtained by TAP-CRNN are encouraging, we will further test the performance using a larger dataset of heart sound in the future.

This study has several limitations. As a major limitation, this study focused on the specific heart sound patterns of VSD, while not considering other types of structural heart diseases. Although heart murmurs can be heard in many other congenital and valvular heart diseases, such as atrial septal defects, patent ductus arteriosus, mitral regurgitation, and aortic regurgitation, patients with these diseases were not included in this study. Harmless heart murmurs, which occasionally occur in normal subjects, were also not included^43–45^. A larger heart sound database is currently being established to comprehensively collect heart sounds from patients with all types of structural heart diseases. An advanced version of the proposed TAP-CRNN algorithm that can recognize the specific murmur types in such diseases is also under development.

## Conclusions

We demonstrated that a TAP-CRNN model can accurately recognize the systolic murmur of VSD patients. As compared to CNN and CRNN without TAP, the proposed TAP-CRNN achieves higher sensitivity and specificity scores for systolic murmurs detections in patients with VSDs. The results suggest that by incorporating the attention mechanism, the CRNN-based model can more accurately detect murmur signals. We also noted that sounds recorded from the second aortic and the tricuspid areas can facilitate more accurate murmur detection results as compared to other auscultation sites. The experimental results from the present study confirmed that the proposed TAP-CRNN serves as a promising model for the development of software to classify the heart murmurs of many other types of structural heart diseases.

## Methods

In this section, we introduce our data source, algorithm, and analysis method.

## Data source

The sound dataset used in this study included heart sounds recorded from subjects at the National Taiwan University Hospital (NTUH) using an iMediPlus electronic stethoscope. This study was approved by the research ethics committee of NTUH, and informed consent was obtained from all subjects or, if subjects are under 18, from a parent and/or legal guardian in accordance with the Declaration of Helsinki. It is also confirmed that all methods were carried out in accordance with relevant guidelines and regulations.

Sounds from patients diagnosed with VSD were categorized as the VSD group and sounds from patients without a significant heart malformation were categorized as a normal group. Auscultation was applied for each subject by a cardiologist with 30 years of experience to confirm whether a pathological systolic murmur occurred in patients with VSD. Normal subjects with innocent murmurs were not included in this study. Echocardiography was conducted on all subjects to confirm the disease diagnosis^46^.

For each subject, two repeated heart sound recordings lasting 10 s each were made at each of the following sites: the aortic area (the second intercostal space on the right sternal border), the pulmonic area (the second intercostal space on the left sternal border), the secondary aortic area/Erb’s point (the third intercostal space on the left sternal border), the tricuspid area (the fourth intercostal space on the left sternal border), and the mitral area/apex (the fifth intercostal space to the left of the midclavicular line)^30,47^. The sounds were recorded by trained study nurses under the supervision of an experienced cardiologist. The soundtracks were saved as WAV files.

The soundtracks collected were divided into training and test sets. Notably, the training and test sets are two mutually exclusive sets without an overlap.

## Algorithm characteristics

**Figure 3:**
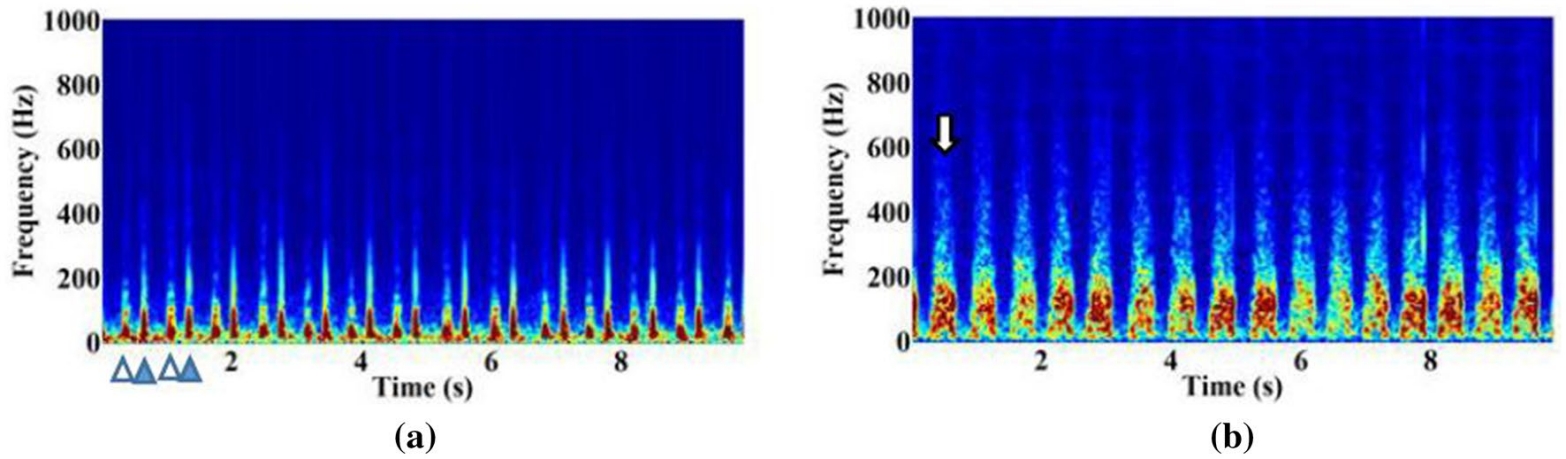
Spectrograms of heart sounds from the normal subjects (**a**) and the subjects with VSD (**b**). The spectrums of sounds or other signals as they vary with time is shown. S1 (empty triangle) and S2 (solid triangle) are observed in the spectrogram of the normal heart sound. Systolic murmur (white arrow) is observed in the spectrogram of the VSD heart sound.

**Figure 4:**
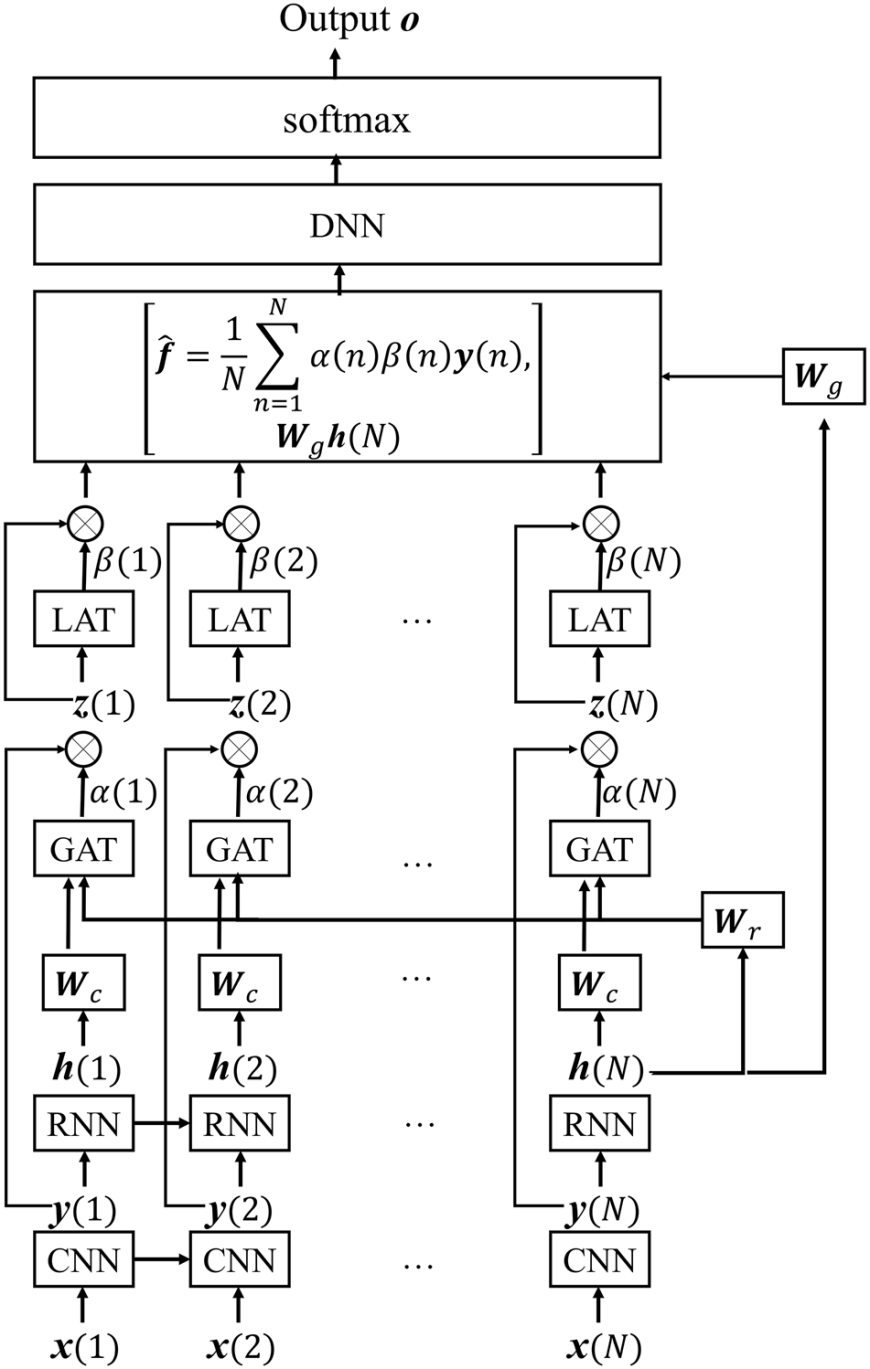
The mechanism of TAP-CRNN.

In this study, a short-time fast Fourier transformation was used to transform the phonocardiogram (PCG) signal into a time–frequency representation (spectral features), where ${\bf{X}} = \left[ {{\varvec{x}}(1) , \ldots , {\varvec{x}}(n), \ldots ,{\varvec{x}}(N) } \right]$ denotes the input feature, and *N* is the number of frames of **X**. Each frame is represented by a 257-dimensional log-power spectral feature vector. The collection of frames in **X** forms a spectrogram, which is generally used to visualize the characteristics of temporal signals varying over time (Fig. 3). In this study, the TAP-CRNN structure was used for classification^27^. Figure 1 shows the network architecture of TAP-CRNN, in which convolutional layers^48^ were used to extract invariant frequency-shift features ${\bf{Y}} = \left[ {{\varvec{y}}(1) , \ldots , {\varvec{y}}(n) , \ldots ,{\varvec{y}}(N) } \right].$ Recurrent layers^49^ were used to explore the global temporal feature ${\varvec{h}}\left( {N} \right)$ of a sequence from the recurrent layer’s outputs, and the TAP layers then extracted the temporal attentive feature and weighed the spectral features when generating the classification results. Figure 4 shows CRNN with a TAP mechanism. The idea here is to focus on important features or regions by introducing attention blocks. Two different attention approaches, local and global, were used to exploit the effectiveness of the TAP mechanism. The back-propagation algorithm is adopted to train the TAP-CRNN parameters to minimize the cross entropy^50^. In terms of global attention, the model decides to focus equally on all regions (global). By contrast, local attention focuses on small regions (local). The idea of global attention is to consider all outputs of the convolutional layer and the temporal summarization of the output of the recurrent layer. For global attention of the TAP, we employ a simple concatenated layer to construct the global attentive vector ***c***(*n*) by combining the information from the output of the convolutional layer ${\varvec{y}}\left( {n} \right)$ and the output of the recurrent layer ${\varvec{h}}\left( {N} \right)$, such as in the following:

1 $${\varvec{c}}\left( {n} \right) = \left[ {\begin{array}{*{20}c} {{\varvec{W}}_{c} {\varvec{y}}\left( {n} \right)} \\ {{\varvec{W}}_{r} {\varvec{h}}\left( {N} \right)} \\ \end{array} } \right],$$

where ${\varvec{h}}\left( {N} \right)$ is the output of the recurrent layer at the last time step, ${\varvec{W}}_{c}$ and ${\varvec{W}}_{r}$ are the parameter matrices used to concatenate ${\varvec{y}}\left( n \right)$ and ${\varvec{h}}\left( {N} \right)$, i.e.,${\varvec{W}}_{c} \in R^{{cnn_{dim} \times cnn_{dim} }}$ and ${\varvec{W}}_{r} \in R^{{rnn_{dim} \times rnn_{dim} }}$, where $cnn_{dim}$ and $rnn_{dim}$ are the output dimensions of the convolutional and recurrent layers, respectively.

The global attentive vector ${\varvec{c}}\left( {n} \right)$ is subsequently fed into the global attention block to produce the global attention weights $\alpha_{global}$ (scalar) and is shown as follows:

2 $$\alpha_{global} \left( {n} \right) = softmax\left( {{\varvec{u}}^{T} \tanh \left( {{\varvec{c}}\left( {n} \right) + {\varvec{b}}_{global} } \right)} \right),$$

where ${\varvec{u}} \in R^{{\left( {cnn_{dim} + rnn_{dim} } \right) \times 1}}$ is the vector used to calculate the global attention weight matrix shared by all time steps, and ${\varvec{b}}_{global} \in R^{{\left( {cnn_{dim} + rnn_{dim} } \right) \times 1}}$ is the global bias matrix. The global attention weights are used to weight the local features from the convolutional layer at each time step as follows:

3 $${\varvec{z}}\left( {n} \right) = \alpha_{global} \left( n \right){\varvec{y}}\left( n \right),$$

In addition to the global attention, the local attention is used to further refine the feature extraction and is calculated in the following manner:

4 $$\beta_{local} \left( n \right) = softmax\left( {{\varvec{v}}^{T} {\text{tanh}}\left( {{\varvec{W}}_{l} {\varvec{z}}\left( n \right) + {\varvec{b}}_{l} } \right)} \right),$$

where ${\varvec{W}}_{l} \in R^{{cnn_{dim} \times cnn_{dim} }}$, ${\varvec{b}}_{l} \in R^{{cnn_{dim} \times 1}}$, and ${\varvec{v}} \in R^{{cnn_{dim} \times 1}}$ are the parametric matrices used for the local attention weight calculation. These local attention weights are used to weight the features such as in the following:

5 $${\varvec{f}}\left( {n} \right) = { }\alpha_{global} \left(n \right) \beta_{local} \left( n \right)\user2{ y}\left(n \right),$$

where $\beta_{local} \left( n \right)$ is the output weight vector for local attention. The final attentive context is calculated as the average of the weighted outputs and is shown as follows:

6 $$\hat{\user2{f}} = { }\frac{1}{N}\mathop \sum \limits_{n = 1}^{N} \alpha_{global} \left( n \right) \beta_{local} \left( n \right)\user2{ y}\left( n \right),$$

After obtaining the attentive context $\hat{\user2{f}}$, we concatenate it with the last time step output ${\varvec{h}}\left( {N} \right)$ of the CRNN as the input ***s*** of the dense layers, such as in the following:

7 $${\varvec{s}} = { }\left[ {\begin{array}{*{20}c} {\hat{\user2{f}}} \\ {{\varvec{W}_g\varvec{h}}\left( {N} \right)} \\ \end{array} } \right]{ },$$

The dense layers are constructed using fully connected units. The relationship between feature ***s*** and the output of the first hidden layer is described as follows:

8 $${\varvec{a}}_{1} = F\left( {{\varvec{W}}_{1}^{{}} {\varvec{s}} + {\varvec{b}}_{1} } \right),$$

where ${\varvec{W}}_{1}$ and ${\varvec{b}}_{1}$ correspond to the weight and bias vector in the first layer*,* and *F*(.) is the activation function. After obtaining the output of the first hidden layer, the relationship between the current and next hidden layer can be expressed as follows:

9 $${\varvec{a}}_{l} = F\left( {{\varvec{W}}_{l}^{{}} {\varvec{a}}_{l - 1} + {\varvec{b}}_{l} } \right), \, l = 2, \ldots ,L,$$

where *L* is the total number of layers of neurons in the output layer. Thus, the relationship for the classification layer or the output layer can be described as follows:

10 $${\varvec{o}} = G\left( {{\varvec{a}}_{L} } \right),$$

where *G*(.) is the softmax function, and ***o*** is the final output of TAP-CRNN.

**Figure 5:**
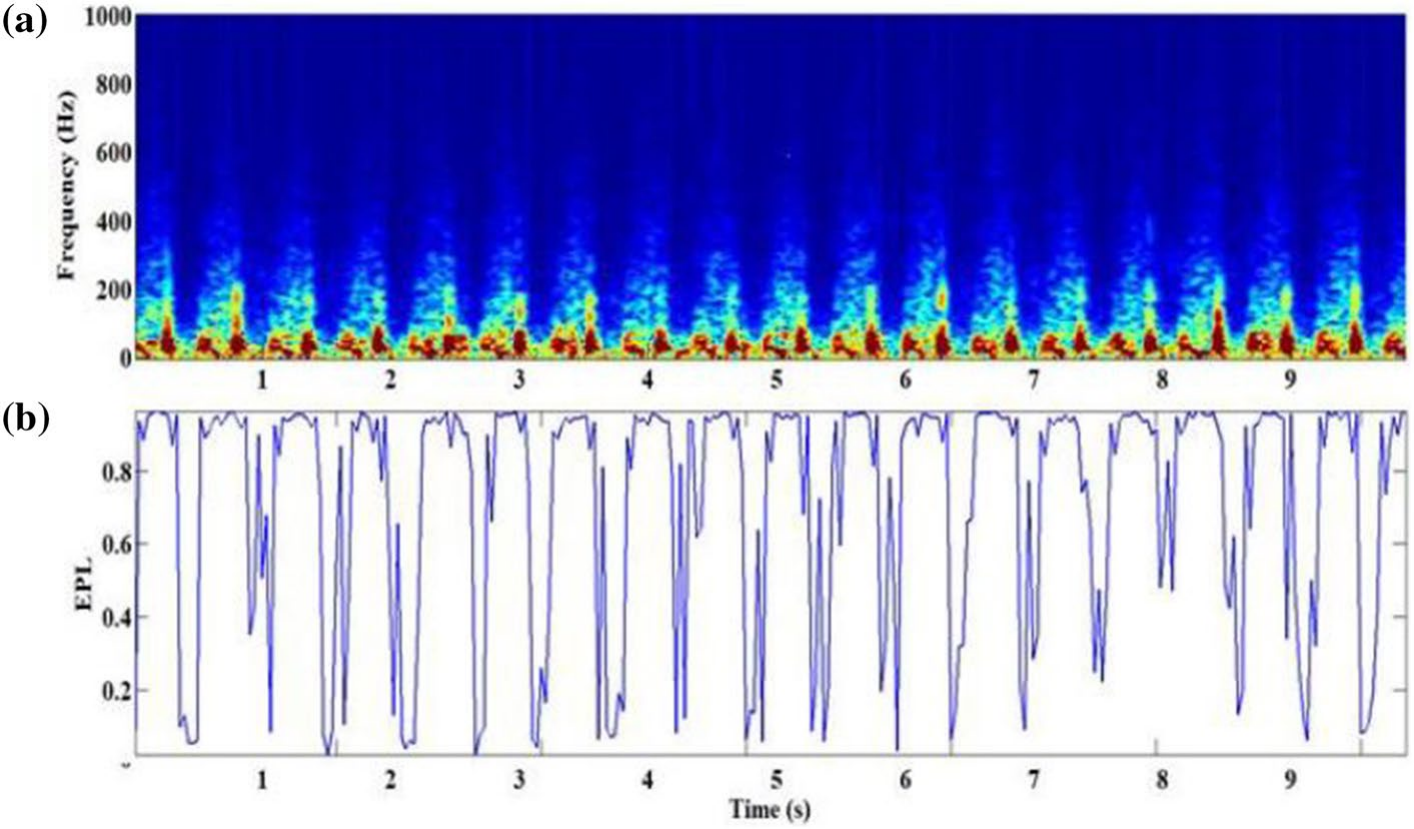
(**a**) The spectrogram of heart sounds from the subjects with VSD, and (**b**) the product of global attention and local attention coefficients.

The importance coefficients provided by the global and local attention were regarded as a frame-based event presence likelihood (EPL), i.e., $\alpha_{global} \left( n \right) \beta_{local} \left(n \right)$. To determine the classified result, the frames with low EPLs were ignored while being emphasized with high EPLs. Figure 5 illustrates the spectrogram (Fig. 5a) and the EPL score (Fig. 5b) of heart sounds from subjects with VSD, in which the murmur regions showed high EPLs when the global attention coefficients and the local attention coefficients were calculated. The features of the murmur regions with a high EPL will be emphasized during the feature extraction.

## Statistical analysis

The sex distribution, mean age, mean height, and mean weight of the subjects were calculated. An independent sample t-test and a chi-square test were conducted to compare the differences between the VSD and normal groups in terms of continuous and categorical variables, respectively.

The soundtracks used in the test set were applied to test the recognition performance. The accuracy, sensitivity, specificity, positive predictive value (PPV), and negative predictive value (NPV) for the distinction of the systolic murmur of VSD patients from the normal heart sounds of healthy volunteers were calculated^51–53^. A diagnosis using echocardiography was applied as the gold standard for these calculations^9,10,54^. The equations are as follows:

11 $$Accuracy = \frac{Tp + Tn}{{Tp + Tn + Fp + Fn}}$$

12 $$Sensitivity = \frac{Tp}{{Tp + Fn}}$$

13 $$Specificity = \frac{Tn}{{Fp + Tn}}$$

14 $$PPV = \frac{Tp}{{Tp + Fp}}$$

15 $$NPV = \frac{Tn}{{Fn + Tn}}$$

where *Tp* indicates a true positive, *Tn* indicates a true negative, *Fp* indicates a false positive, and *Fn* indicates a false negative.

## Supplementary information

Supplementary Information.
