# Supplementary material for: Automatic recognition of murmurs of ventricular septal defect using convolutional recurrent neural networks with temporal attentive pooling
Source: Sci Rep. 2020 Dec 11;10:21797. doi: 10.1038/s41598-020-77994-z (PMC7732853; doi:10.1038/s41598-020-77994-z)
Supplement: Supplementary file 1 — Supplementary Information. [file 41598_2020_77994_MOESM1_ESM.pdf]

Appendix

Supplemental Table 1.

Positive and negative events in the CNN model to recognize systolic murmur and normal heart sounds.

	Echocardiographic diagnosis	
	VSD	Normal
**CNN model test results**		
Murmur-like	156	9
Normal-like	22	51

Supplemental Table 2.

Positive and negative events in the CRNN model to recognize systolic murmur and normal heart sounds.

	Echocardiographic diagnosis	
	VSD	Normal
**CRNN model test results**		
Murmur-like	163	4
Normal-like	15	56

Supplemental Table 3.

Positive and negative events in the TAP-CRNN model to recognize systolic murmur and normal heart sounds.

	Echocardiographic diagnosis	
	VSD	Normal
**TAP-CRNN model test results**		
Murmur-like	172	1
Normal-like	6	59

Supplemental Table 4.

Positive and negative events in the TAP-CRNN model to recognize systolic murmur and normal heart sounds in the aortic area.

	**Echocardiographic diagnosis**	
	VSD	Normal
**TAP-CRNN model test results**		
Murmur-like	42	1
Normal-like	2	11

Supplemental Table 5.

Positive and negative events in the TAP-CRNN model to recognize systolic murmur and normal heart sounds in pulmonic areas.

	**Echocardiographic diagnosis**	
	VSD	Normal
**TAP-CRNN model test results**		
Murmur-like	32	0
Normal-like	2	12

Supplemental Table 6.

Positive and negative events in the TAP-CRNN model to recognize systolic murmur and normal heart sounds in the second aortic area.

	**Echocardiographic diagnosis**	
	VSD	Normal
**TAP-CRNN model test results**		
Murmur-like	33	0
Normal-like	0	12

Supplemental Table 7.

Positive and negative events in the TAP-CRNN model to recognize systolic murmur and normal heart sounds in the tricuspid area.

	Echocardiographic diagnosis	
	VSD	Normal
**TAP-CRNN model test results**		
Murmur-like	33	0
Normal-like	0	12

Supplemental Table 8.

Positive and negative events in the TAP-CRNN model to recognize systolic murmur and normal heart sounds in the mitral area.

	Echocardiographic diagnosis	
	VSD	Normal
**TAP-CRNN model test results**		
Murmur-like	32	0
Normal-like	2	12
